# ‘Navigating the uncharted waters’- Transition experiences of novice advanced practice nurses in China: a descriptive phenomenological study

**DOI:** 10.1186/s12912-026-04626-8

**Published:** 2026-04-06

**Authors:** Wei Tan, Qin Hu, Shanshan Liu, Cong Wang, Lei Wang, Yan Jiang

**Affiliations:** 1 Evidence-based Nursing Center, West China Hospital, Sichuan University/West China School of Nursing, Sichuan University, Chengdu, China; 2 Department of Nursing, West China Hospital, Sichuan University/West China School of Nursing, Sichuan University, Chengdu, China; 3 Sichuan Provincial Engineering Research Center of Medical Nursing Equipment and Materials, Chengdu, China

**Keywords:** Advanced practice nurses, Experience, Qualitative study, Transition

## Abstract

**Background:**

Transitioning to the role of advanced practice nurses is dynamic and challenging. Deeply exploring the transition experience and developing corresponding intervention strategies can make the transition smoother. China’s development of advanced practice nursing remains at a nascent stage, exhibiting localized characteristics. However, limited knowledge exists regarding experiences, feelings, and challenges of advanced practice nurses in the region. This study explored the transition experiences of novice advanced practice nurses in China.

**Methods:**

A descriptive phenomenological qualitative design was used. Twenty-three advanced practice nurses were recruited using purposive sampling technique. Data were collected using face-to-face interviews and analysed using thematic analysis.

**Results:**

“Navigating the uncharted waters” was the overarching theme, which linked four themes: (1) being constrained by the practical environment; (2) reconstructing professional identity; (3) bearing multiple burdens; and (4) steering my own course. The study found that, notwithstanding institutional barriers and an unfamiliar environment, these participants actively reconstructed their professional identities to meet evolving expectations, shouldered workload, interpersonal, and psychological burdens, and steered their own course through the initial transition phase. Furthermore, distinctive characteristics were identified among Chinese advanced practice nurses, specifically the phenomenon of “disguise” in their work practice and a strong aspiration for a graduate degree.

**Conclusions:**

This study reveals that the role transition for advanced practice nurses is a complex and demanding process, profoundly shaped by the practical environment. The findings further highlight the importance of adaptive coping behaviours, particularly personal resilience and proactive learning. Healthcare institutions, nursing organizations, and national policymakers should offer strong support by enhancing educational programs and developing transition plans, thereby creating a positive environment for advanced practice nurses. Future longitudinal research should explore the experiences of advanced practice nurses throughout various career stages and identify the factors that influence their professional trajectories.

**Trial registration:**

Our study does not report healthcare interventions on human participants. Clinical trial number: not applicable.

**Supplementary Information:**

The online version contains supplementary material available at 10.1186/s12912-026-04626-8.

## Introduction

The International Council of Nurses defines an advanced practice nurse(APN) as “a generalist or specialized nurse who has acquired the expert knowledge base, complex decision-making skills and clinical competencies for advanced nursing practice” [1]. To address the dual challenges of the continuous increase in healthcare demands and the ongoing shortage of healthcare professionals, the development of APNs has exhibited a global trend [2]. Currently, approximately 70 countries or regions have introduced APNs [3], and are at different stages of developing advanced practice nursing [4]. The value of APNs has been widely demonstrated. The evidence is substantial that APNs contribute to providing high-quality healthcare services [5], improving patient clinical outcomes and satisfaction [6], and reducing medical costs [7].

The development and implementation of new roles is never an easy task, and the transitional phase is particularly crucial [8]. For APNs newly entering the workforce, the transition to this new role is a dynamically changing and multi-challenging process, requiring them to master new knowledge and skills, shoulder new responsibilities, establish new professional relationships, and integrate into new practice areas [9]. Multiple international studies have shown that novice APNs encounter difficulties and obstacles, including low personal confidence, a lack of recognition from other healthcare professionals, and heavy work burdens [10]. These difficulties often lead to adverse consequences, such as negative emotions, low job satisfaction, job burnout, and even increased intention to leave [11]. It is evident that the transition to APNs is problematic [12]. A comprehensive understanding of the transition and looking at changes in transition can provide deeper insights into the “fuzzy logic of practice”, which is very helpful for facilitating the transition [13]. For example, a study constructed a theoretical model “from Limbo to Legitimacy” through qualitative interviews, describing common practice experiences in the transition to the primary care nurse practitioner role [14]. On the basis of this common experience, scholars subsequently organized targeted webinars, which were shown to positively influence the NP’s perceptions of role transition [15]. Therefore, deeply exploring the transition experience and developing corresponding intervention strategies can make the transition smoother.

Internationally, given the importance of the transition experience, many scholars have conducted a series of studies on this topic, identifying some common phenomena and analysing related obstacles and promoting factors [16]. However, many participants in these studies have held the role of APNs for a long time, almost more than 1 year [17]. For example, a qualitative study of nurse practitioners who have been working in the Netherlands for 2 to 6 years revealed that they have achieved success and that role transition is driven by ambitions [18]. Existing research has primarily focused on their post-transition experiences and professional growth, while paying less attention to the transition process between old and new roles—particularly their initial clinical experiences after completing educational training programs. This may be attributed to the long recall period of the participants, as some of their early feelings may have faded from memory.

As an innovative role that expands the boundaries of traditional nursing practice, the development of APNs in mainland China is still in the nascent stage. In the early 21st century, scholars began to explore this topic, but their discussions were limited to theoretical research, focusing on translating and analysing foreign concepts. Since 2010, the education and training of APNs, such as curriculum design, training objectives, and core competencies, has become a research hotspot [19]. Subsequently, several medical schools and healthcare institutions initiated pilot education programs [20]. In 2017, the Peking University School of Nursing enrolled students in the Nurse Practitioner Master’s program in Chronic Disease Management [21]. In 2021, the West China Hospital of Sichuan University launched an in-service training program for APNs [22]. However, few practical studies exist, and few scholars have focused on the experiences and performance of APNs after they enter the workforce. Only one study has described the work experiences of APNs in the ICU, finding that they have unclear professional positionality and are prone to role conflicts [23]. There may be a disconnect between education and actual clinical practice, highlighting the need for further practical exploration to optimize training content and develop effective transition programs.

Additionally, China’s social culture, healthcare system, and health service needs differ from those of other countries. There are also differences in nurses’ social status, educational background, and staffing levels [24]. Although the Chinese Nursing Association has proposed starting an APN program, APNs have not yet been protected by law, and public awareness of their role is limited [25]. Therefore, APNs in mainland China follow a localized model, and their experiences may differ from those in other countries. Moreover, no Chinese scholars have yet researched the transition to APNs, making the exploration of this field even more urgent in mainland China. Therefore, this study explored the transition experiences of novice APNs in China and provide insights for developing educational programs and intervention strategies to support smooth transition.

## Methods

### Study design

A descriptive phenomenological qualitative design was used. This method helps to obtain comprehensive and direct descriptions of participants’ experiences [26]. The reporting of this study adhered to the Consolidated Criteria for Reporting Qualitative Research (COREQ) checklist [27].

### Setting

This study was conducted in two tertiary hospitals in southwest China, both recognized as pilot sites for the APN role, with certified APNs deployed across multiple departments.

The APN training program referenced in this study was based on a nationally developed core competency framework encompassing six domains: clinical care, consultation, coordination, leadership, teaching, and research [28]. The resulting curriculum comprised 36 modules (12 h each). Certification required passing both theoretical assessments and an Objective Structured Clinical Examination (OSCE), administered by the participating hospitals. Within these hospitals, APN responsibilities varied by clinical department but generally focused on key areas: interprofessional collaboration, complex patient care, early identification of acutely deteriorating patients, nurse-led clinic consultations, and collaborative prescribing. In addition, APNs contributed to clinical teaching and evidence-based practice initiatives.

These hospitals were selected because they represent early APN implementation sites in China, ensuring participants had completed training. Furthermore, departmental diversity provided rich context for examining APNs’ transition experiences.

### Sampling method and procedure

Purposive sampling was employed to recruit participants from two tertiary hospitals in mainland China in July 2023. The inclusion criteria were: (1) completion of the APN training curriculum; (2) current employment as an APN; and (3) less than one month of experience in the APN role, to capture the initial phase of role transition. The exclusion criteria were: (1) were on leave during the data collection period; and (2)inability or unwillingness to provide informed consent.

The nursing departments of these hospitals provided APN rosters. Based on these rosters, researchers purposively selected and contacted potential participants by telephone to explain the study’s purpose and procedures. Only those who expressed willingness to participate were included in the final sample.

#### Demographic characteristics of the sample

Twenty-three participants aged between 32 and 50 years participated in this study. The majority of the participants were female (*n* = 21), and held a bachelor’s degree (*n* = 18). The demographic characteristics are presented in Table 1.

**Table 1:** Demographic characteristics of the participants (*n* = 23)

Characteristics	Frequency(*n*)	Percentage(%)
Sex		
Male	2	8.70
Female	21	91.30
Age		
31–40	13	56.52
41–50	10	43.48
Marital status		
Married	23	100.00
Single	0	0
Level of Education		
Bachelor	18	78.26
Master	5	21.74
Nursing tenure (Years )		
9–14	4	17.39
15–20	8	34.78
21–26	11	47.83
Practice area		
Biological therapy	1	4.35
Cardiovascular Surgery	1	4.35
Endocrinology	2	8.70
Endoscopy	2	8.70
Gastroenterology	1	4.35
General surgery	4	17.39
Geriatrics	3	13.04
Hemodialysis	1	4.35
Lung cancer	1	4.35
Nephrology	1	4.35
Orthopedics	1	4.35
Rehabilitation medicine	2	8.70
Thoracic Surgery	2	8.70
Urology	1	4.35

### Data collection and management

A semi-structured interview guide was developed based on a literature review and team discussions. Two pilot interviews were conducted with APNs from one of the participating hospitals to assess the clarity and relevance of the questions; these interviews were excluded from the final analysis [29]. Based on feedback from the pilot interviews, the interview guide was revised and improved, resulting in a final version comprising eight open-ended questions (Supplementary File 1).

Between September and December 2023, the first author conducted individual face-to-face interviews using the interview guide in a quiet hospital conference room [30], encouraging participants to freely share their experiences [31]. Written informed consent was obtained from all participants prior to each interview. With participants’ permission, all interviews were audio-recorded using a portable recorder. During the interviews, probes were used to elicit more detailed responses [32]. The first author maintained a reflexive journal to document personal assumptions, potential biases and thoughts [33]. Data saturation was achieved after the 21st interview, as no new information emerged [34]. Two additional interviews were conducted to confirm saturation, resulting in a final sample of 23 participants. Interviews lasted 31 to 103 min (mean: 64 min).

The audio recordings were transcribed verbatim within 24 h of the interviews. All audio recordings, transcripts, and related documents were stored on a password-protected computer accessible only to the research team.

### Data analysis

An inductive thematic analysis was conducted following Braun and Clarke’s six-phase framework: familiarising yourself with your data, generating initial codes, searching for themes, reviewing themes, defining and naming themes, and producing the report [35]. Two authors read and reread the transcripts for coding and then collated the codes into potential themes and subthemes. Regular team meetings were held to discuss, refine, and reach consensus on the emerging themes. During these meetings, we also critically examined our assumptions and positionalities to ensure the reflexivity of the analysis. NVivo 12.0 software was used as an organizational tool to support systematic coding and theme development.

### Rigor

Rigor in qualitative research refers to the trustworthiness of the research process and findings, including four criteria: credibility, transferability, dependability and confirmability [36]. To maintain rigor, this study adopted several trustworthiness strategies. All the interviews were conducted by the same author to maintain consistency and ensure data credibility. She is a PhD student in nursing with research experience in advanced nursing practice and qualitative studies, has a strong interest in the topic, and does not have any apparent biases or professional relationships with the participants. In addition, all interviews were audio-recorded and transcribed systematically to ensure credibility. A comprehensive presentation of the context, participants, and data collection and analysis procedures and a detailed description of the findings were provided to establish transferability. Dependability was ensured by using field notes and sharing transcribed data and preliminary analysis results with the participants. This process allowed participants to confirm that their experiences were accurately represented [37]. Two authors separately coded the transcription data and held regular meetings with other team members, further increasing the confirmability of the data.

## Results

**Fig. 1:**
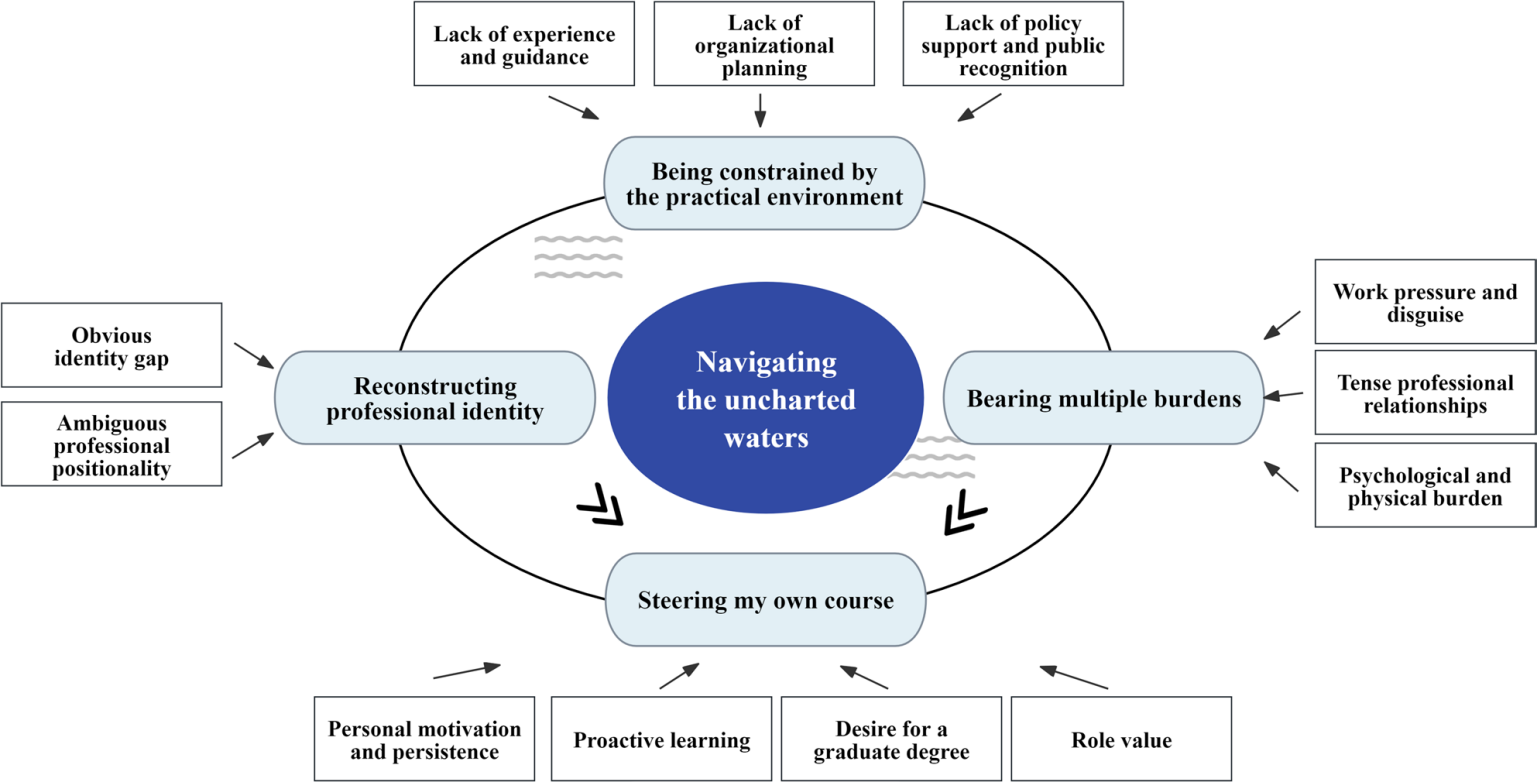
Overview of themes and sub-themes

During the data analysis process, a total of 456 initial codes were generated and categorized into 38 categories. These categories were further synthesized into 4 themes and 12 sub-themes. An overarching theme, “Navigating the uncharted waters,” emerged as the central concept linking all themes, reflecting the state of exploration and adjustment in an unfamiliar and uncertain terrain of novice APNs. The four main themes identified were: (1) being constrained by the practical environment; (2) reconstructing professional identity; (3) bearing multiple burdens; and (4) steering my own course (Fig. 1).

### Theme 1: Being constrained by the practical environment

This theme encompasses the multifaceted environmental barriers that novice APNs encountered as they attempted to establish their roles and deliver advanced nursing care. Three key dimensions of constraint were identified: (1) lack of experience and guidance at the individual level, (2) lack of organizational planning at the institutional level, and (3) lack of policy support and public recognition at the systemic level.

#### Lack of experience and guidance

This sub-theme captures the challenges arising from the pioneering nature of the APN role in China. As one of the first cohorts of APNs in their institutions, participants found themselves navigating uncharted professional territory with no established templates or experienced colleagues to guide them.

One participant complained:

*“There is no template for us to follow in China owing to the lack of studies and experience. Therefore*,* we should learn from foreign models and internalize something.” (N11)*.

Another participant echoed this sentiment, expressing the frustration of exploring independently:

*“I feel lost*,* exploring on my own without sufficient support. In foreign countries*,* mentorship is prevalent*,* but as one of the first batches of APNs in China*,* I don’t have a mentor.” (N06)*.

#### Lack of organizational planning

A key barrier identified was the lack of organizational planning, manifested through limited learning resources, workforce instability, and mismatched compensation. Participants reported that these factors collectively limited their ability to realize their professional potential.

Some participant noted the scarcity of development opportunities:

*“The institution doesn’t provide us with adequate resources*,* such as opportunities to attend academic conferences or participate in teaching and presentation activities.” (N06)*.

Another participant described how frequent staff changes disrupted their work environment:

*“For various reasons*,* many young doctors have resigned from the department*,* creating a turbulent environment that complicates my work.” (N09)*.

Compensation misalignment further undermined motivation, with one participant reflecting:

*“With so much work pressure*,* my salary has not increased*,* which undermines my motivation.” (N10)*.

#### Lack of policy support and public recognition

Almost all participants identified the absence of national policy support as a significant barrier, as it meant the APN role title lacked legal protection and formal recognition. They underscored how this regulatory gap constrained their scope of practice and diminished their authority in clinical settings.

*“Currently, my work is restricted because there is no legislation for APNs in China (mainland)*,* even in Sichuan Province. I feel like I don’t have the authority to direct others.” (N06)*.

Some participants encountered situations where patients were confused by their title and responsibilities, which further undermined their confidence.

*“The employee badge of the APN is different from that of other nurses. Once*,* a patient asked*,* ‘Why does your badge look different? Are you from another hospital?’ This indicates that they aren’t familiar with the role of APNs.” (N15)*.

### Theme 2: Reconstructing professional identity

Central to this theme was the significant shift participants experienced in their professional identity. Many participants felt caught between their past roles and new roles. This identity tension was further complicated by the ambiguous positionality of their role in their clinical departments, where they did not belong to any groups. Two subthemes were identified: (1) obvious identity gap; and (2) ambiguous professional positionality.

#### Obvious identity gap

A key finding was that the transition from experienced registered nurse to APN often felt like a regression from “expert” to “novice.” This obvious identity gap left many participants struggling to adapt.

*“It’s like going from being an expert in one role to a novice in another. I’m thrust into an unknown world*,* with an inconspicuous identity.” (N10)*.

*“The APN is a completely new clinical role*,* and this transition to a new role and novice identity has left me feeling somewhat unsettled. I haven’t adjusted yet.” (N08)*.

This identity gap was compounded by the mismatch between participants’ initial aspirations and their actual practice. Some participants expressed frustration when their ambitions did not translate into clinical progress.

*“After a long period of course learning*,* I felt ambitious. But then I didn’t make any progress in clinical practice. There is a gap between my expectations and my actual work performance. I have a bit of regret.” (N22)*.

#### Ambiguous professional positionality

Several participants described that their professional position as awkward and ill-defined. They found themselves occupying an ambiguous space—recognized as advanced practitioners, yet lacking a clearly defined place within their clinical departments. This ambiguity left many participants feeling isolated.

One participant used a metaphor to describe this situation:

*“We all feel that we are in an awkward position. What is an APN? The title is so prominent that it seems like we are at the top of the mountain*,* but in fact we are at the foot of the mountain.”(N18)*.

Another participant explained how this ambiguous positioning affected her daily work:

*“After becoming an APN*,* I was marginalized and felt like I didn’t belong to any group in the department. My position cannot be classified as administrative*,* as I possess no substantive managerial authority; nor does it fall under clinical nursing*,* since I am no longer responsible for specific patients…I’m just a bystander.”(N06)*.

Unclear professional boundaries added another layer of difficulty. One participant described how overlapping responsibilities led to conflict:

*“The difference between my role and other roles is not obvious*,* so role overlap often occurs: the head nurse and I both manage critically ill patients*,* and the quality control nurse and I both oversee care quality. This has led to role conflict*,* as they feel I’ve overstepped the boundary and encroached on their authority.” (N09)*.

### Theme 3: Bearing multiple burdens

This theme reflects the multiple burdens participants encountered as they stepped into their new roles. These included the difficulty of fulfilling advanced responsibilities, the pressure to meet performance expectations, the tensions in interactions with colleagues. Collectively, these challenges negatively impacted on their psychological and physical well-being. Three sub-themes were identified: (1) work pressure and disguise; (2) tense professional relationships; and (3) psychological and physical burden.

#### Work pressure and disguise

Some participants reported that the shift in their work practices, combined with the challenges of taking on more advanced duties, created significant stress for them. To meet departmental performance evaluation requirements, some found it necessary to temporarily “disguise” their role by taking on a large number of routine tasks.

*“These advanced tasks are still very difficult for me. Therefore*,* I have to complete more basic tasks to disguise*,* such as evaluating interns and checking nursing records*,* which help me meet the workload requirements set by the department manager. ” (N14)*.

One participant even described herself as an‘odd-job’ person:

*“I’m now an ‘odd-job’ person*,* filling in for others when needed. When the nursing group leader is on leave*,* I take her role. Sometimes*,* the head nurse assigns me a lot of clerical work: sort documents*,* attend meetings…….Although these tasks are not fully aligned with my professional role expectations*,* they contribute to creating an appearance of substantial workload.” (N08)*.

#### Tense professional relationships

The transition to advanced role disrupted participants’ established professional relationships. Many participants perceived a change in the way their colleagues related to them, even leading to tension.

Collaborating with physicians proved particularly challenging. One participant recalled her hesitation when communicating clinical issues:

*“When I find issues in clinical practice*,* I will alert doctors*,* but sometimes they don’t believe me*,* which makes me feel timid and hesitant.” (N02)*.

Some participants also struggled to gain acceptance from their nursing colleagues. Working beyond traditional nursing boundaries, they were met with misunderstanding. One participant reflected on this experience:

*“Some nurses gossip that ‘APNs don’t belong to clinical nursing groups*,* become ‘officials’*,* sit in ‘offices’*,* and work effortlessly.’ I’m not sure if this is out of jealousy*,* but it makes me feel sad and lost.” (N19)*.

#### Psychological and physical burden

This sub-theme captures the emotional and physical toll of navigating an unfamiliar and demanding role. Many participants described feelings of uncertainty, self-doubt, and hopelessness in the face of professional setbacks.

One participant expressed her frustration after a failed collaboration:

*“A week ago*,* I tried to collaborate with surgical departments on blood sugar management for patients undergoing major abdominal surgeries*,* but it fell through. Now*,* I feel depressed and hopeless about the future.” (N09)*.

Another participant emphasized the anxiety of expanded responsibilities:

*“Previously*,* as a nursing group leader*,* I was responsible for the patients in my group only. Now*,* I should pay attention to all patients in the ward*,* which often leaves me feeling doubtful and unconfident.” (N10)*.

These emotional struggles extended beyond psychological burden, manifesting in physical symptoms such as disrupted sleep and unhealthy eating patterns.

*“I have an emotional eating problem*,* tending to eat a lot when I’m feeling down*,* especially after work.” (N09)*.

*“In the first one or two weeks of being employed as APN*,* I often lost sleep.”(N19)*.

### Theme 4: Steering my own course

Despite encountering numerous difficulties, participants actively sought ways to navigate their own careers. Driven by personal motivation and persistence, they engaged in proactive learning, pursued further education, and gradually discovered the role value. Four subthemes were identified: (1) personal motivation and persistence; (2) proactive learning; (3) desire for a graduate degree; and (4) role value.

#### Personal motivation and persistence

The intrinsic motivation and persistence helped participants cope with adversities and overcome professional challenges. Many participants drew motivation from the sense of fulfillment they gained through clinical practice.

*“When I solve clinical problems for patients*,* I feel a sense of fulfilment. This is also why I choose to be an APN - to immerse myself in clinical practice and develop my expertise.” (N08)*.

One participant described how persistence shaped her approach:

*“I believe that internal persistence is the most crucial element*,* as it drives one to respond proactively. When facing communication challenges*,* I seek the assistance of the head nurse to facilitate my communication with the doctor.” (N07)*.

#### Proactive learning

A key strategy for navigating the transition was proactive learning. Participants actively strengthened their expertise within their specialty while expanding knowledge across disciplines to better manage patients with complex conditions.

*“I attended formal training to master the skills of ultrasound examinations. Now*,* in my department*,* doctors ask me to perform bedside ultrasound examinations for patients after kidney biopsy.” (N12)*.

*“I continue to expand my knowledge in multidisciplinary areas.”(N16)*.

Several participants attributed their proactive learning to a perceived gap between educational training and the specialty-specific demands of clinical practice. Thus, proactive and in-depth learning was necessary to achieve advanced nursing practice.

*“The APN training program focuses on critical thinking and methodology*,* teaching me how to systematically solve clinical problems. However*,* there remains a deficiency in specialty specific depth. When encountering complex patient cases in clinical*,* I find it necessary to pursue additional learning.”(N12)*.

Learning also facilitated participants’ integration into their departments. Some participants used learning-derived activities such as organizing case discussions and sharing clinical experiences to build connections with colleagues.

*“I regularly collect challenging patient cases from the ward and organize case discussions to achieve common progress.” (N08)*.

#### Desire for a graduate degree

A strong desire for postgraduate education was evident among participants, who viewed a graduate degree as a key factor in advancing both personal and professional development.

Several participants were actively preparing for postgraduate entrance examinations, dedicating considerable time and effort.

*“I devote more time to preparing for the graduate admission test and also attend training courses on weekends.’ (N2)*.

One participant currently pursuing graduate studies described the demands of balancing academic and clinical work:

*“Combining graduate studies with clinical work keeps me very busy*,* I am eager to obtain a graduate degree.” (N7)*.

Participants articulated multiple motivations for pursuing a graduate degree. Some looked to international models, noting the trend toward higher education requirements for APNs.

*“In many countries*,* the postgraduate degree is mandatory for APNs. Although this requirement has not yet been implemented in China*,* it represents a future trend. ” (N4)*.

Some participants emphasized the professional authority associated with advanced degrees.

*“In clinical settings*,* practitioners with higher educational qualifications are typically accorded greater professional authority.” (N9)*.

Institutional incentives also played a role, as one participant noted:

*“For promotion system in the hospital*,* a postgraduate degree is an indispensable prerequisite.” (N5)*.

#### Role value

A recurring finding was participants’ gradual recognition of their value. Some participants characterized their roles as the “glue” that held multidisciplinary teams together, the “warners” who identified patient risks, and the “instructors” who guided clinical nurses.

*“I communicate with respiratory therapists and dieticians*,* discuss the outcomes of these communications with doctors*,* and formulate relevant plans. The doctors often take my advice. Now I act as the ‘glue’ between them.” (N02)*.

*“After rescuing a patient*,* I would reflect on why the patient’s condition had changed and why such a treatment plan was adopted and consult relevant literature. Then*,* I communicate with the doctor to sort out the procedure for rescuing such patients*,* provide guidance to other nurses*,* and supervise them in following the procedure.” (N04)*.

For other participants, recognition of their value stemmed from external feedback. Even without significant clinical achievements, positive responses from colleagues validated their efforts.

*“I feel so proud when the doctor asks me something. As I received positive feedback.” (N02)*.

## Discussion

This study represents the first attempt to explore transition experiences for APNs in mainland China, with a specific focus on novices who had been in practice for less than one month. The core finding reveals that these APNs navigated uncharted waters of their new roles. Constrained by the complexities of an unfamiliar environment, they undertook efforts to reconstruct professional identities, managed multiple responsibilities, and actively steered their own courses through this transitional phase. This study enhances our understanding of the transition of novice APNs and provides valuable insights for optimizing educational models and developing transition programs.

From a macro perspective, the practical environment refers to China’s political, cultural and social background. First, the lack of policy support is the greatest issue at present. Owing to the short history of developing APNs in mainland China, relevant health policies have not yet been introduced to define their rights and obligations, resulting in a lack of protection for the title. Concurrently, limited public awareness channels for APNs and the absence of official and authoritative information have led to low recognition. This finding has been confirmed in several studies in China [25, 38].

It can be seen that at the policy level, a legal and regulatory framework that supports the development of advanced practice nursing is necessary. However, such systemic transformation can not be achieved overnight [39]. It requires a stepwise implementation of pilot programs to demonstrate the significance and value of APNs within China’s healthcare system. Due to the pioneering nature of APNs in China, there is limited practical research and scarce experience. As the first exploratory qualitative study in this field in China, while focusing on APNs’ individual experiences, this research has revealed their environmental challenges and provided preliminary evidence of their clinical contributions. These findings lay the groundwork for developing a standardized national system for APN training, certification, and regulation in China.

From a micro perspective, the practical environment refers to institutions or departments where APNs work. We found that organizational characteristics, such as high staff turnover, a discordant atmosphere, and the inequitable treatment of APNs by the organization, such as mismatched incentives and unbalanced learning opportunities, significantly hindered the role transition of APNs. These contextual constraints contribute to more negative experiences.This phenomenon may be particularly pronounced in mainland China, where APNs are regulated at the institutional level, thereby amplifying the impact of work environment [40]. Our findings suggest that healthcare institutions, as the primary governing bodies of APN practice, should provide adequate support to maximize their professional potential.

The transition in professional identity resulted in a sense of gap, which was not favourable in the early stage. Shifting from “expert” to “novice,” from “familiar” to “unknown,” APNs felt that they had returned to the starting point of their career [41], facing even more severe challenges than novice registered nurses with no prior knowledge, due to the unclear boundaries and responsibilities of their professional roles. These findings are consistent with those of previous studies [8, 42].

“Bystander”, “awkward”, “marginalized”, and “odd-job” were their descriptions of themselves, as their role had not yet been differentiated from other clinical roles. They found themselves caught between multiple responsibilities, leading to role overlap and potential role conflict. This ambiguous professional positionality exacerbated tensions within the workplace, such as scepticism from physicians and isolation from their nursing colleagues [43]. Compared with previous studies [9, 44], the process of professional identity reconstruction in this study seems to be even more demanding. This may be due to our participants’ limited tenure (less than one month), which enabled them to provide more profound insights into the challenges encountered during the initial transition phase. Participants in previous studies had generally completed their role transition, and their reflections tended to focus on the overall role changes [45].

Our finding is consistent with other studies showing that APNs bear multiple burdens [46]. However, the sources of these burdens differ from those identified in previous studies [18, 41]. APNs find it challenging to dedicate time during work hours for deep thinking and planning because they do not want to be misinterpreted as “idle” by other nurses and instead have to “disguise” themselves with multifarious workloads. This is also related to tense work relationships. Faced with doubts from colleagues, they lack the confidence to take on challenging tasks and tend to choose more basic and error-free tasks. This kind of “disguise” seems to be a unique manifestation among APNs in China, which may conceal their talents.

As repeatedly mentioned in previous studies, negative emotions, including “anxious”, “frustrated”, “depressed”, and “annoyed”, occur frequently in APNs [47, 48]. Insomnia and emotional eating are unique findings in this study, further highlighting the challenges and frustrations faced by APNs, particularly those with short employment durations. These findings suggest that support strategies are urgently needed.

Proactive learning is currently the simplest and most effective coping strategy available to APNs, particularly in acquiring interdisciplinary knowledge and skills. This may be related to the current core professional positioning of Chinese APNs - solving complex multidisciplinary clinical problems. In countries such as the United States, The United Kingdom, and Singapore, the educational system for nurse prescribing is well-developed [49], with prescriptive authority established as a hallmark function of APNs [50]. However, in mainland China, there is a cautious attitude towards nurse prescribing [51], and most APNs are not yet able to regard prescription decision-making as a career breakthrough [52]. Instead, they tend to focus on complex case management and multidisciplinary collaboration, thus forming a unique professional advantage. Therefore, the education of APNs should not be limited to cognitive development and theoretical methodologies but should also incorporate clinical interdisciplinary modules [53]. For practicing APNs, it is essential to provide multidisciplinary communication platforms and support resources, such as interdisciplinary case discussions, peer support systems, and mentorship programs.

A master’s degree is recommended for entry level by International Council of Nurses (ICN) [1]. And this criterion has been widely adopted in countries with well-established advanced practice nursing systems, such as the United States, United Kingdom, and Canada [54]. The guideline published by ICN also indicates that the characteristics of APNs are shaped by the context in which they are credentialed to practice. For example, a review of APNs in Low- and lower middle-income countries revealed that the type of education ranged from continuing education and non-accredited post-registration diploma to masters [3]. Therefore, viable approaches for developing and implementing new APN role must be grounded in each country’s specific healthcare system and nursing workforce [55, 56]. Currently in China, only 0.2% of nurses hold graduate degrees [57], which presents significant challenges in directly applying the international master’s degree standard. Therefore, selecting experienced bachelor’s-degree nurses as potential APN candidates is a feasible strategy during the initial phase of role implementation [22].

Although not all APNs currently hold master’s degrees, this study reveals a strong aspiration for graduate education among them. This phenomenon can be attributed to multiple factors: higher educational qualification can enhances professional confidence and authority, fosters recognition within interdisciplinary teams, and increases probability of professional title advancement - collectively leading to better alignment with APN role expectations. This creates a virtuous cycle of “education- APN role”: the APN role motivates nurses to pursue higher education, while the attainment of higher education further enhances role congruence. These findings substantiate the positive impact of APN role implementation on the professional development of nursing.

### Strengths and limitations

This is the first study to explore the transition experience of novice APNs within mainland China, offering some perspectives that differ from those of previous research conducted primarily in Western countries. In addition, including participants of different genders, ages, educational backgrounds, and departments contributes to the richness of the research data. However, there are several limitations in this study. An interview is a form of self-report, which may involve recall bias [58]. Therefore, we selected APNs who had been employed for less than a month to minimize recall bias and directly revealed the conflicts and issues encountered during the early transition period. Moreover, this study was conducted in a city, so the generalizability of our findings may be limited. However, this is due to the small number of APNs in China, and it is also designed to achieve face-to-face and in-depth interviews to obtain the information conveyed by facial expressions and body language.

### Implications of the study

The findings underscore the need for structured institutional support, including clear professional scope, mentorship programs, and performance evaluation aligned with advanced practice responsibilities, to facilitate novice APNs’ transition into practice. They also highlight the importance of strengthening APN curricula with specialty-specific content to bridge the gap between education and clinical demands. At the policy level, regulatory frameworks need to be gradually developed to formally define the APN role and provide legal protection. Collectively, these insights point to the need for longitudinal research to examine APNs’ experiences across different stages of transition and to evaluate how such interventions shape their professional development.

## Conclusion

This study makes novice APNs’ roles and experiences more transparent. Being constrained by the practical environment, reconstructing professional identity, and bearing multiple burdens were identified as three main themes, which illustrated a grim picture of the APNs’ experiences. However, despite the tumultuous initial phase of transitioning into a new role, they steered their own course, with some even realizing their professional value. Additionally, we observed unique characteristics among APNs in China, including “disguise” in their work and a strong desire for a graduate degree. Future longitudinal research is needed to examine how APNs’ experiences evolve across career stages, serving as a foundation for optimizing educational models and developing targeted support programs.

## Supplementary Information

Below is the link to the electronic supplementary material.

Supplementary Material 1

## Data Availability

The dataset and analysis supporting the conclusions of this article is available under request to the corresponding author.
