# Supplementary material for: ‘Navigating the uncharted waters’- Transition experiences of novice advanced practice nurses in China: a descriptive phenomenological study
Source: BMC Nurs. 2026 Apr 6;25:507. doi: 10.1186/s12912-026-04626-8 (PMC13234987; doi:10.1186/s12912-026-04626-8)
Supplement: Supplementary file 1 — Supplementary Material 1 [file 12912_2026_4626_MOESM1_ESM.docx]

**‘Navigating the uncharted waters’- Transition experiences of novice advanced practice nurses in China: A descriptive phenomenological study**

**Supplementary File 1: The interview guide.**

| **Interview stage** | **Primary questions** |
| --- | --- |
| Opening question | - What were your experiences during the transition from a registered nurse to an advanced practice nurse? How did you feel? |
| Continued question | - How was your overall mood in the past one months? |
|  | - What personal growth have you experienced since becoming an advanced practice nurse? What are your shortcomings? |
|  | - Could you describe how your work has changed? For exa mple, in terms of your work responsibilities and work patterns? |
|  | - How did the new role affect your personal life? |
|  | - What challenges did you face? What did you do to deal with them? |
|  | - What support have you received? How did you find the available support? What additional support do you need? |
| Closing question | - Is there anything else you would like to share about your transition experience? |
